# Reduced type 2 diabetes incidence reflecting end of post‑World War II calorie restrictions in Germany. Reply to Nilsson PM, Vaag A [letter]

**DOI:** 10.1007/s00125-024-06255-9

**Published:** 2024-08-17

**Authors:** Carolin T. Lehner, Gunther Schauberger, Stefanie J. Klug

**Affiliations:** Chair of Epidemiology, TUM School of Medicine and Health, Technical University of Munich, Munich, Germany

**Keywords:** Epidemiology, Famine, Germany, Incidence rates, Time trend, Type 2 diabetes

*To the Editor*: We cordially thank Professors Nilsson and Vaag [1] for their comments on our study [2] and the interesting discussion they initiated. We are pleased to see their acknowledgement of and interest in our study [2]. In their letter, Nilsson and Vaag [1] hypothesise that calorie restriction during or post World War II may have influenced the incidence of type 2 diabetes in older age groups in Germany between 2012 and 2021. We agree that previous research has shown some evidence that the undernutrition of women during pregnancy may be a risk factor for type 2 diabetes in their offspring [3, 4].

In response to Nilsson and Vaag’s letter [1], first, our data do not allow further investigation of this hypothesis. The data used in our analysis were aggregated in 10 year age groups (Table 1) and we are therefore unable to analyse the effect of birth year on incidence of type 2 diabetes, for example to help explain why the decline in type 2 diabetes incidence was steeper between 2012 and 2016 than between 2017 and 2021 [2].

**Table 1:**
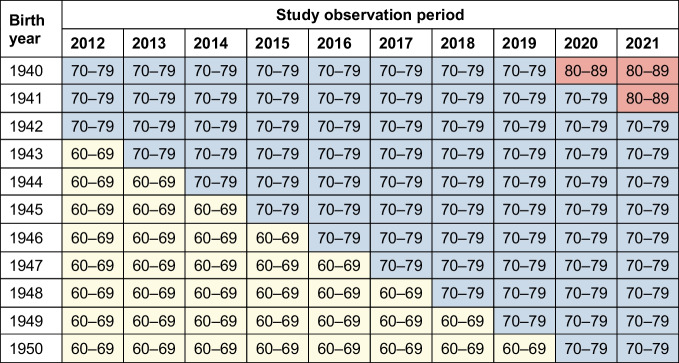
Allocation of available 10 year age groups to birth years between 1940 and 1950 for each year of our observation period 2012–2021

Second, we would like to take the opportunity to share our opinion on certain aspects of the discussion initiated by Nilsson and Vaag [1]. Their main hypothesis is that a contributing factor to the decrease in type 2 diabetes incidence observed between 2012 and 2021 for the age groups 50 years and older might be exposure to famine in utero during and shortly after World War II. For illustration using our data, Table 1 displays the allocation of our 10 year age groups to birth years between 1940 and 1950 for each year of our observation period 2012–2021. Table 1 shows that exposure to famine in Germany during and after World War II (1940–1950) would, at most, affect the two age groups 60–69 years and 70–79 years. For the age group 60–69 years, the share of the birth cohort 1940–1950 decreases from 2012 to 2021, while, for the age group 70–79 years, the share of the birth cohort 1940–1950 increases from 2012 to 2021. Therefore, for the age group 70–79 years, an increase in incidence of type 2 diabetes between 2012 and 2021 would have been expected if exposure to famine in utero had had a major influence. All other age groups would not be affected by this hypothesis; however, our age-stratified analysis shows that the decrease in incidence of type 2 diabetes between 2012 and 2021 was similar for the age groups 50–59, 60–69, 70–79, 80–89 and 90–120 years (see Fig. 2 in [2]).

Overall, we see no evidence in our data that calorie restriction during and after World War II is reflected in the observed decreasing trend in type 2 diabetes incidence rates between 2012 and 2021 in the age groups 50–59, 60–69, 70–79, 80–89 and 90–120 years in our secondary analysis of routinely collected German health claims data.
